# A DNA Vaccine against Yellow Fever Virus: Development and Evaluation

**DOI:** 10.1371/journal.pntd.0003693

**Published:** 2015-04-13

**Authors:** Milton Maciel, Fábia da Silva Pereira Cruz, Marli Tenório Cordeiro, Márcia Archer da Motta, Klécia Marília Soares de Melo Cassemiro, Rita de Cássia Carvalho Maia, Regina Célia Bressan Queiroz de Figueiredo, Ricardo Galler, Marcos da Silva Freire, Joseph Thomas August, Ernesto T. A. Marques, Rafael Dhalia

**Affiliations:** 1 Johns Hopkins University, School of Medicine, Department of Pharmacology & Molecular Sciences, Baltimore, Maryland, United States of America; 2 Oswaldo Cruz Foundation (FIOCRUZ), Aggeu Magalhães Research Centre, Department of Virology, Laboratório de Virologia e Terapia Experimental (LAVITE), Universidade Federal de Pernambuco (UFPE), University City, Recife, Pernambuco, Brazil; 3 Health Secretariat of the State of Pernambuco, Central Public Health Laboratory-LACEN, Boa Vista, Recife, Pernambuco, Brazil; 4 Oswaldo Cruz Foundation (FIOCRUZ), Oswaldo Cruz Institute, Bio-Manguinhos, Laboratório de Tecnologia Virológica (LATEV), Manguinhos, Rio de Janeiro, Brazil; 5 Federal Rural University of Pernambuco, Department of Veterinary Medicine, Dois Irmãos, Recife, Pernambuco, Brazil; 6 University of Pittsburgh, Center for Vaccine Research, Pittsburgh, Pennsylvania, United States of America; Institute of Tropical Medicine (NEKKEN), JAPAN

## Abstract

Attenuated yellow fever (YF) virus 17D/17DD vaccines are the only available protection from YF infection, which remains a significant source of morbidity and mortality in the tropical areas of the world. The attenuated YF virus vaccine, which is used worldwide, generates both long-lasting neutralizing antibodies and strong T-cell responses. However, on rare occasions, this vaccine has toxic side effects that can be fatal. This study presents the design of two non-viral DNA-based antigen formulations and the characterization of their expression and immunological properties. The two antigen formulations consist of DNA encoding the full-length envelope protein (p/YFE) or the full-length envelope protein fused to the lysosomal-associated membrane protein signal, LAMP-1 (pL/YFE), aimed at diverting antigen processing/presentation through the major histocompatibility complex II precursor compartments. The immune responses triggered by these formulations were evaluated in H2b and H2d backgrounds, corresponding to the C57Bl/6 and BALB/c mice strains, respectively. Both DNA constructs were able to induce very strong T-cell responses of similar magnitude against almost all epitopes that are also generated by the YF 17DD vaccine. The pL/YFE formulation performed best overall. In addition to the T-cell response, it was also able to stimulate high titers of anti-YF neutralizing antibodies comparable to the levels elicited by the 17DD vaccine. More importantly, the pL/YFE vaccine conferred 100% protection against the YF virus in intracerebrally challenged mice. These results indicate that pL/YFE DNA is an excellent vaccine candidate and should be considered for further developmental studies.

## Author Summary

DNA and other nucleic acid vaccine technologies are advancing quickly, and new potent delivery methods are demonstrating great potential in human clinical trials. In this manuscript, we report a highly protective DNA vaccine against the yellow fever virus. This vaccine was engineered with a molecular adjuvant technology to enhance the exposure of the vaccine antigens to the immune system, resulting in augmented CD4^+^ helper responses. We postulate that the robust CD4^+^ responses help the B cells and the CD8^+^ cells mature more efficiently and produce better antibodies and cytotoxic cells, respectively. Our results show that vaccination with this yellow fever DNA formulation elicited protective levels of neutralizing antibodies and very strong cellular responses at similar levels to the responses elicited by the live attenuated 17DD vaccine. In addition, these results also suggest a very important role for cellular responses in mediating protection against yellow fever virus. The results reported here are very promising and further studies may lead to a new yellow fever vaccine for human use.

## Introduction

The yellow fever (YF) virus is considered the prototype member of the family *Flaviviridae*, which includes several other viruses of medical importance, such as the dengue, Japanese encephalitis, tick-borne encephalitis and West Nile viruses [1]. According to the World Health Organization (WHO), more than 200,000 cases of YF infection, including 30,000 deaths, occur annually, with 90% of cases occurring in Africa [2]. The safest strategy for preventing YF infection is still vaccination because there is currently no drug that is effective against YF virus infection. In the last 70 years, more than 500 million people around the world have been vaccinated with the YF 17D/17DD virus-attenuated vaccines with a remarkable record of safety and efficacy [3]. Attenuated YF virus vaccines generate both long-lasting neutralizing antibodies and T-cell responses [4, 5]. However, despite several improvements in the manufacturing process and quality control, severe side-effects resulting from vaccination continue to be reported [6–9]. In some cases, vaccination was associated with increased severity of symptoms [10] and on rare occasions with fatal reactions [11, 12]. In view of this, the development of alternative vaccination strategies, such as DNA-based vaccines encoding specific virus sequences, has been considered [13–16].

The YF virus genome consists of a single-stranded, positive-sense RNA molecule of ~10.8 kb, flanked by a 5’ cap and a 3’ non-polyadenylated terminal loop structure. It expresses three genes for structural proteins (capsid—C, pre-membrane/membrane—pM/M, and envelope—E) and seven genes that code for non-structural (NS) proteins (NS1, NS2a, NS2b, NS3, NS4a, NS4b, and NS5). Coexpression of flavivirus M and E genes in mammalian cells has been demonstrated to produce virus-like particles (VLPs) containing pM/M and E proteins [17–19]. The E protein is known to be the principal virus surface protein and the main target for neutralizing antibodies. pM/M and E coexpression, as a vaccination strategy, has been described as a way of triggering neutralizing antibodies against the Japanese encephalitis [19–21], West Nile [22] and dengue viruses [17,18,23,24].

DNA vaccines express endogenous cytoplasmic antigens, which are mostly introduced to the immune system through the major histocompatibility complex (MHC) class I molecules that are mostly associated with cellular cytotoxic responses and often fail to elicit a satisfactory humoral response, which is essential for efficient virus neutralization. Activation of CD4^+^ helper cells is important for the development of CD8^+^ responses, immunological memory [25], antibody maturation, class switching and expansion of antigen-specific B cells [26]. Several strategies have been proposed for enhancing MHC class II presentation of antigens encoded by DNA vaccines. The targeting of the MHC II compartment with other flavivirus E antigens has been shown to enhance neutralizing antibody production in immunized mice [17, 22, 24] and in non-human primates (Raviprakash personal communication at 2004 ASTMH meeting, http://www.astmh.org/meeting_archives.htm).

One of the main strategies for targeting the MHC II compartment with DNA-encoded antigens is based on the expression of the antigen fused to the lysosomal-associated membrane protein 1 (LAMP-1), a protein primarily found in the outer membrane of lysosomes [27]. The chimeric antigens expressed by DNA formulations in the context of type I trans-membrane LAMP are directed to compartments rich in MHC II, called the MHC II compartment (MIIC), which is where the peptide-MHC II complexes are formed [28, 29]. Other LAMP/antigen chimeric strategies, such as LAMP/HIV Gag [25, 26, 30, 31] and LAMP/dengue virus 2 pM/M-E [17, 24] antigens, have been shown to target the MIIC and were found to elicit enhanced immune responses compared with vaccines encoding unmodified native antigens.

This study investigated T-cell and humoral immune responses to the envelope of YF virus in C57Bl/6 and BALB/c mice immunized with DNA formulations expressing the full-length YF envelope protein, either as a wild-type or fused to LAMP. Responses in the mice were compared with the results obtained with standard immunization using the YF 17DD vaccine. We also evaluated the ability of DNA vaccines to provide protection against a lethal challenge. We show that although the YF 17DD vaccine produced higher neutralizing antibody titers, both DNA vaccine constructs encoding the entire E protein were also able to protect the mice against lethal challenge.

## Materials and Methods

### Yellow fever vaccine, cells and antibodies

The attenuated 17DD human yellow fever vaccine was obtained from Bio-Manguinhos, a unit of the Oswaldo Cruz Foundation (FIOCRUZ, Rio de Janeiro, Brazil). The vaccine was reconstituted in chilled PBS, kept in an ice bath, and used for mouse immunizations within 4 hours of reconstitution. VERO and 293 cells were obtained from the ATCC (Rockville, MD, USA) and were grown according to the supplier’s instructions in a DMEM medium (Invitrogen) containing 10% fetal bovine serum (Gibco), 1% penicillin/streptomycin (Gibco) and 1% L-glutamine (Sigma). YF virus strain 17DD was propagated in Vero cells at 37°C in 5% CO_2_ to a titer of 10^6^ plaque-forming units (PFUs) per ml. The polyclonal anti-YF hyperimmune serum used in immunofluorescence assays was obtained from mice immunized with the YF 17DD virus-attenuated vaccine in our laboratory. Secondary antibodies were purchased either from Jackson Immunoresearch Laboratories (Bar Harbor, ME, USA) or Molecular Probes (Seattle, WA, USA).

### Peptides

A set of 120 peptides of 15 amino acids each (15-mers), overlapping by 11 amino acids (15x11) and comprising the entire length of the envelope protein of the YF 17DD virus (NCBI GenBank accession number U17066), was synthesized using Schafer-N (Copenhagen, Denmark). The peptides were HPLC-purified to 80% purity or greater, with the exception of a few peptides that could not be purified and were used as crude extracts. The identity of each peptide was confirmed via mass spectrometry, and the amount of purified peptide was precisely measured. Stock solutions of all peptides were prepared via dilution in water when possible, or in a solution of 10 to 100% DMSO, to a final concentration of 20 mg/mL and were stored at −20°C. For the ELISPOT assays, the peptides were used at a 10 μg/mL final concentration. The highest DMSO concentration in the ELISPOT experiments was 0.05%.

### DNA constructs

**Fig 1:**
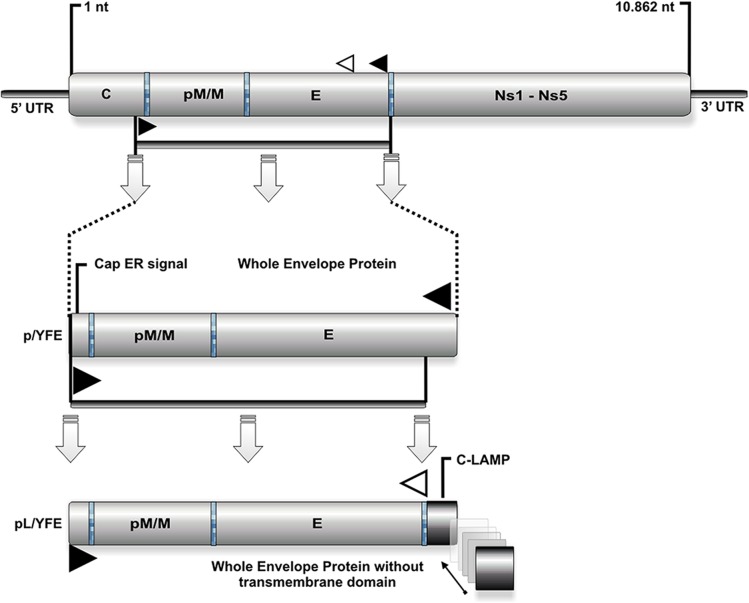
p/YFE and pL/YFE DNA vaccine construct design. The fragment pM/M-E (extending from nucleotides 392 to 2452, black arrows) was amplified using PCR and cloned into a p43.2 vector to generate the p/YFE vector. This construct starts with the ER capsid signal and ends with the envelope trans-membrane domain. To generate the pL/YFE construct, we designed a second forward primer that annealed just upstream of the envelope trans-membrane domain to amplify the fragment extending from nucleotides 392 to 2323. This fragment was fused to the C-terminal end of LAMP and cloned into the same vector.

The full YF genome, used as template to design primers for p/YFE and pL/YFE amplification, is deposited in NCBI’s GenBank under accession number NC 002031. The Kyte-Doolittle hydropathy plot analyzed this sequence to identify the capsid ER translocation signal and the predicted envelope trans-membrane domain of the YF genome. The wild-type pM/M-E amplicon starts with the ER capsid signal and ends with the envelope trans-membrane domain. To generate the pL/YFE construct, we designed a reverse primer that hybridizes to the YF genome just upstream of the envelope trans-membrane domain to replace it with the human membrane anchor and cytoplasmic domains of LAMP (**Fig 1**). The DNA pM/M-E sequence was amplified from the YF 17DD infectious clone using specific primers that incorporated an ATG start site in the context of the Kozak sequence and a translational stop codon. PCR amplification was performed using the proofreading TGO DNA polymerase (Roche, Indianapolis, IN, USA) and 0.6 μM of each primer. The amplicon was inserted into the p43.2 vector between the *Xho*I and *Not*I cleavage sites to generate the p/YFE construct. The pL/YFE construct, however, was obtained in two steps. First, the pM/M-E sequence was amplified using a reverse primer that hybridized upstream of the trans-membrane domain of the YF envelope protein. Then, the PCR product was inserted into the p43.2 vector between the *Nhe*I and *Xho*I sites, generating an intermediate construct (p/YFE_INT_), ready to receive the membrane anchor and cytoplasmic domains of LAMP. Second, LAMP was amplified from the p43.2-Gag/LAMP vector and was inserted into p/YFE_INT_, between the *Xho*I and *Xba*I sites, to generate the pL/YFE construct. Both the p/YFE and pL/YFE constructs were checked by sequencing; among the 2,061 nucleotides of the pM/M-E wild-type construction (p/YFE) that encodes 687 amino acids, two nonsynonymous mutations were found. An alanine (A) was replaced with a valine (V) at position 250, and a serine (S) was replaced with an aspartic acid (D) at position 349. Given that both mutations were also found at the same locations in the 644-residue sequence of the pL/YFE construction, they are very likely present in the YF 17DD infectious clone that was used as a DNA template. Regardless of the source of the two mutations, both mutations were deemed to be irrelevant for our vaccine studies as the E protein has several B-cell and T-cell preserved epitopes distributed along its sequence.

### Transfections, western blotting and fluorescence assays

293 cells were plated onto cover slips and transfected with p/YFE, pL/YFE or empty p43.2 vectors, using Lipofectamine 2000 (Invitrogen Life Technologies). For Western blotting, transfections were carried out in 6-well tissue culture plates with 10 μg of each plasmid and 40 μl of Lipofectamine 2000, whereas transfections for fluorescence assays were carried out in 24-well tissue culture plates with 2.5 μg of each plasmid and 10 μl of Lipofectamine 2000, both in accordance with the manufacturer’s instructions. Vero cell extracts infected with the YF 17DD virus strain were used as a positive control. After 48 hours, both transfected and infected cell extracts were processed.

For Western blot analysis, cell extracts were resuspended in 2x Laemmli denaturing protein sample buffer, fractionated in 12.5% SDS-PAGE and transferred to a polyvinylidene difluoride (PVDF) membrane. After blocking with 5% milk/0.05% PBS-Tween 20, membranes were incubated for 1 hour with the appropriate primary polyclonal antibodies (anti-YFV hyperimmune rabbit serum, previously produced in our laboratory) diluted 1:500 in 1% milk/0.1% PBS-Tween 20. Membranes were washed 3 times with 1x PBS for 10 minutes/wash and incubated for 1 hour with 1:5,000 goat anti-rabbit IgG antibody conjugated with horseradish peroxidase (Jackson Immunoresearch Laboratories). The Western blot reactions were detected using enhanced chemiluminescence (ECL) reactions (Millipore). For fluorescence assays, cell extracts were fixed in 100% methanol at—20°C for 5 minutes, blocked with 1% BSA/PBS solution for 30 minutes, and incubated with an anti-YFV hyperimmune mouse antibody diluted 1:200 for 1 hour, followed by a 1-hour incubation with secondary antibody diluted 1:500 (Alexa 488-conjugated goat anti-mouse, Molecular Probes, Seattle, WA, USA). The cover slips were then mounted on glass slides using ProLong Gold (Molecular Probes, Seattle, WA, USA) and observed through a confocal microscope. The images were acquired using a Leica SPII-AOBS confocal microscope (Leica Microsystem, Hm) with a 63× oil immersion objective NA 1.3. The Alexa 488 fluorochrome was excited using an ArKr laser at 488 nm. The digital image was acquired using Leica software in a 24-bit RGB format with a 1024 × 1024 pixel area. Fields were chosen for imaging based on the spread and morphology of the cells.

### Immunological assays

Female BALB/c (H2^d^) and C57Bl/6 mice (H2^b^), aged 6 to 8 weeks (Charles River, Kingston, NY, USA), were used for the ELISPOT assays. They were housed in micro-isolator cages under specific pathogen-free conditions and handled in accordance with the Johns Hopkins Institutional Animal Care and Use Committee (IACUC) protocol number MO05M336. The animals were immunized at days zero and 21 and used for the experiments seven to ten days after the last immunization. For the neutralization and protection assays, three-week-old female BALB/c and C57Bl/6 mice were obtained from the Oswaldo Cruz Foundation Breeding Center (Rio de Janeiro, Brazil) and were housed at the Experimental Animal Laboratory (Oswaldo Cruz Foundation, Rio de Janeiro, Brazil) under specific pathogen-free conditions and handled in accordance with the Oswaldo Cruz Foundation Commission for Ethical Animal Use (CEAU) protocol number P0112-02. The animals were immunized at days zero, 30 and 45, and sera were collected via a cut in the tail vein a day before every immunization. For both protocols, the animals were immunized subcutaneously at the base of the tail with either the YF 17DD vaccine at 10^4^ PFUs/50 μl, the DNA constructs at 50 μg/50 μl, or 50 μl of PBS as a negative control.

ELISPOT assays were performed to quantify IFN-gamma spot-forming cells (SFCs) generated via DNA construct immunization. Seven to 10 days after the last immunization, the mice were sacrificed and their spleens were removed. Splenocytes were isolated using standard methods, and single-cell suspensions, depleted of red blood cells, were prepared from freshly isolated splenocytes in culture medium (RPMI 1640 medium supplemented with 10% v/v fetal bovine serum, 100 units/ml penicillin/streptomycin, 2 mM L-glutamine, 50 μM 2-mercaptoethanol and 1 M HEPES buffer). IFN-gamma ELISPOT assays were performed in accordance with the manufacturer’s instructions (BD-Biosciences, San Diego, CA, USA). First, the ELISPOT plates were coated with anti-IFN-gamma antibody at 5 μg/ml and incubated at 4°C overnight. The plates were blocked with RPMI 1640 medium containing 10% FBS for 2 h at room temperature, and total splenocytes (1×10^6^ cells/well) from immunized mice were then added. The cells were cultured at 37°C in 5% CO_2_ with culture medium alone (RPMI 1640 medium supplemented with 5% v/v fetal calf serum, 100 units/ml penicillin/streptomycin, and 2 mM L-glutamine) or with culture medium in the presence of concanavalin A (2.5 μg/ml; Sigma), 10^9^ PFUs/mL of inactivated YF virus as a positive control (strain 17DD), or individual 15-mers from the envelope protein of the YF 17DD virus at 1 μg/ml. After 16 h of culture, the plates were washed and incubated with biotinylated anti-IFN-gamma for 2 h at room temperature, followed by HRP-conjugated avidin for 1 h at room temperature. Reactions were developed with AEC substrate (Calbiochem-Novabiochem Corporation, San Diego, CA, USA). The quantification of spot-forming cells (SFCs) was carried out using the Immunospot Series Analyzer ELISPOT reader (Cellular Technologies Ltd (CTL), Shaker Heights, OH, USA) with the aid of Immunospot software 3.0 (Cellular Technologies Ltd). The data are represented as the number of SFCs/10^6^. The results were considered positive if the number of SFCs was greater than 20 and higher than the background (culture with medium alone) plus three standard deviations. The results are presented after subtraction of the background.

### Viral neutralization assays

Plaque reduction neutralization tests (PRNT) were carried out using VERO cells seeded at a density of 62,500 cells/cm^2^ in 96-well microplates, as previously described [32]. The PRNT tests for the detection of anti-YF nAb were performed after two-fold serial dilutions of serum (1/5 to 1/640) on microtiter plates and incubation with 30 PFUs of the YF 17DD challenge virus strain in each well. After incubation at 37°C in a 5% CO_2_ atmosphere for 1 h, 50 μl of Vero cell suspensions (4×10^4^/well) in medium 199 (Invitrogen) was added, and the plates were incubated at 37°C for 3 h. The medium was then discarded and the cells were overlaid with 100 μl of medium containing 3.5% carboxymethylcellulose. After 6–7 days of incubation at 37°C in 5% CO_2_, cell monolayers were fixed with formalin and stained with crystal violet so plaques could be counted. Standard sera of known antibody content in terms of International Units (IU) were included in each set of tests. The log_10_ dilution of the test and standard sera, which reduced the plaque numbers by 50% relative to the virus control, were determined via interpolation. The mean antibody content at the 50% end point of the standard was then calculated and added to the log_10_ end point for each sample to give log_10_ mIU/ml. Plaque neutralization titers were calculated as the highest dilution of antibody able to reduce 50% of the plaques from input virus. The lower limit of detection of the assay was 84.5 mIU/mL.

### Protection assays and statistical analysis

Groups of three-week-old BALB/c and C57Bl/6 mice immunized three times with either the YF 17DD vaccine or the DNA constructs were inoculated intra-cerebrally with 30 μl of M199 medium containing 10^5^ PFUs of the YF 17DD virus 15 days after the last immunization. The animals were monitored for 21 days and deaths were recorded. Moribund animals were sacrificed by exposure to CO_2_.

Comparisons between ELISPOT and neutralization assay results were made using an unpaired *T*-test. The mean survival times in each group of mice were compared using a one-way analysis of variance (ANOVA) and the Kruskal-Wallis non-parametric test. Statistical tests and graphs were performed and produced using GraphPad Prism version 4.0 (GraphPad Software, San Diego, CA, USA; www.graphpad.com).

## Results

### The p/YFE and pL/YFE DNA constructs express yellow fever proteins in 293 cells

**Fig 2:**
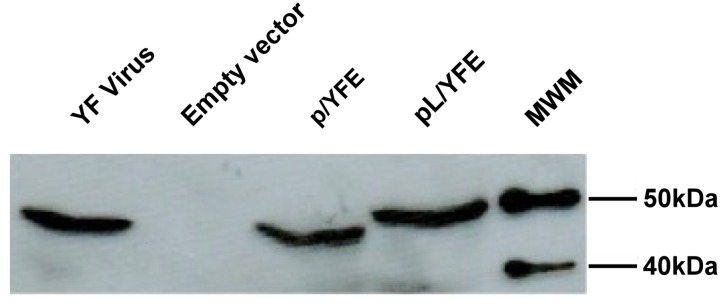
p/YFE and pL/YFE DNA vaccine expression analysis via Western blotting. 293 cells were transfected with the p/YFE or pL/YFE DNA construct and incubated for 48 hours prior to total protein sample preparation. The p43.2 empty vector was used as a negative control, and cells infected with the YF 17DD virus strain were used as a positive control. Cell extracts were transferred to the PVDF membrane and incubated with anti-YFV hyperimmune antibody, followed by incubation with a secondary antibody conjugated with horseradish peroxidase that was revealed using an enhanced chemiluminescence (ECL) reaction. Both the E- and E-LAMP-encoded antigens were successfully expressed and were each the appropriate molecular size.

**Fig 3:**
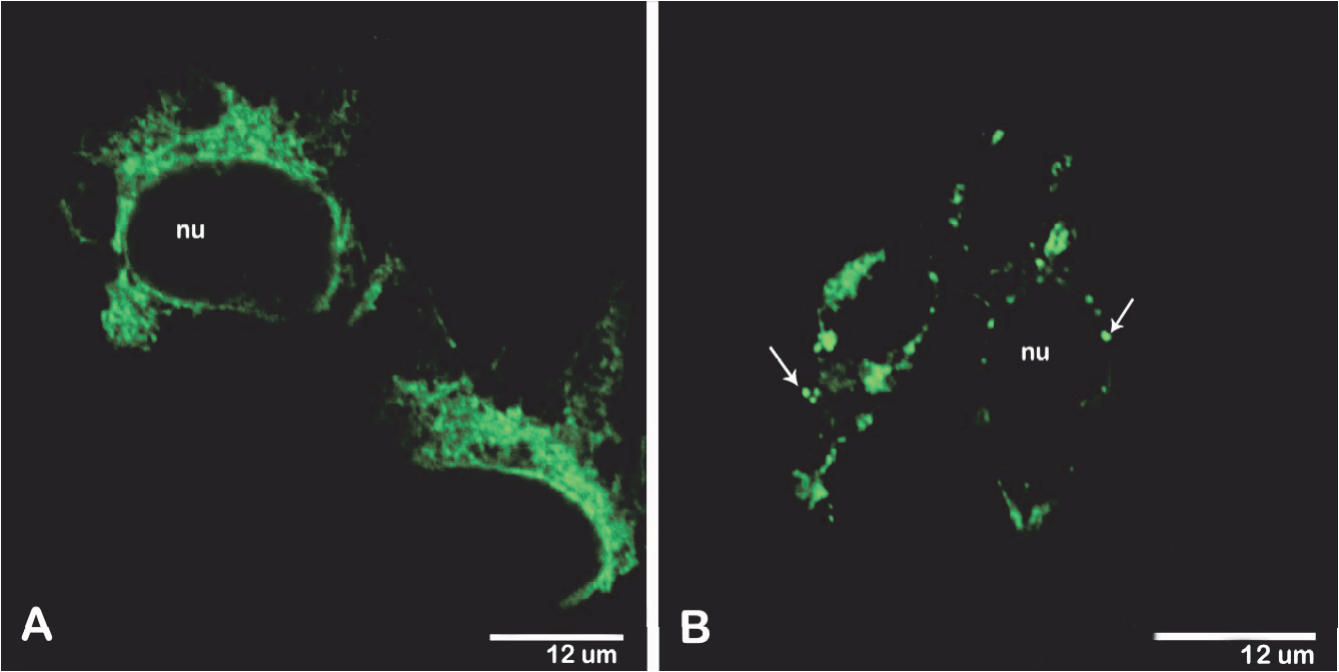
p/YFE and pL/YFE DNA vaccine intracellular steady-state localization analysis using immunofluorescence assays. 293 cells were transfected with the p/YFE or pL/YFE DNA construct and incubated for 48 hours prior to methanol fixation. The p43.2 empty vector was used as a negative control, and cells infected with the YF 17DD virus strain were used as a positive control. Fixed cells were incubated with anti-YFV hyperimmune antibody, followed by incubation with a secondary antibody conjugated with Alexa Fluor-488 dye for microscopic analyses. The characteristic reticular membrane distribution of the viral envelope protein was detected in the p/YFE-transfected cell expressing the wild-type E protein (A), whereas the E/LAMP chimeric protein expressed by the pL/YFE-transfected cells showed the typical punctuated lysosomal-like distribution of endogenous LAMP (B).

293 cells were transfected with the p/YFE and pL/YFE expression vectors encoding the pM/M-E and pM/M-E/LAMP proteins, respectively. Validation of plasmid protein expression and cellular steady-state localization was carried out using Western blot and immunofluorescence analyses. Both the E and E/LAMP proteins were stained using polyclonal anti-YF hyperimmune serum. Western blotting detected specific bands for the E (p/YFE) and E/LAMP (pL/YFE) proteins, as well as the wild-type (YF virus) E protein (**Fig 2**). Immunofluorescence assays showed the characteristic reticular membrane distribution (associated with the typical cellular trafficking of the viral envelope protein) in p/YFE-transfected 293 cells expressing the wild-type E protein (**Fig 3A**). By contrast, the E/LAMP chimeric protein from pL/YFE-transfected cells showed the typical punctuated lysosomal-like distribution of endogenous LAMP (**Fig 3B**). The figure represents several independent assays where the expression and cellular steady-state localization of both the E and E/LAMP proteins were considered to be invariable.

### Characterization of the cellular response triggered by 17DD vaccination in C57Bl/6 mice

It is known that some mouse strains with distinct genetic backgrounds, when exposed to the same antigens, can polarize towards T-helper-1 (Th1) or T-helper-2 (Th2) responses. Some strains are more prone to produce Th1 responses, whereas others are more prone to polarize towards Th2. BALB/c and C57Bl/6 mice strains have been known to produce this type of distinct T-helper responses in several models and, thus, we selected these two strains to investigate how they would respond to our vaccines. An optimized YF 17DD vaccine immunization protocol and IFN-gamma ELISPOT assay conditions, which were previously described for BALB/c (H2^d^: D^d^, K^d^, L^d^, I-A^d^, and I-E^d^) mice [33], were used to characterize the T-cell responses to peptides of the YF 17DD virus proteins in C57Bl/6 (H2^b^: D^b^, K^b^ and I-A^b^) mice. The first round of experiments with total splenocytes led to the identification of 11 antigenic 15-mer peptides from the YF envelope protein. The subsequent experiments were performed using splenocytes depleted of CD4^+^ or CD8^+^ lymphocytes, which lead to the identification of epitopes presented by MHC class I or II, respectively. The depletion typically removed >95% of the targeted population, as assessed via flow cytometry. The CD4-depleted splenocytes, which correspond to the CD8^+^ lymphocyte response, reacted to seven peptides, whereas the CD8-depleted splenocytes, which correspond to the CD4^+^ lymphocyte response, were able to respond to five 15-mer peptides (**Table 1**). The results of quantitative (IFN-gamma SFCs/10^6^) epitope mapping for the YF envelope protein in 17 DD immunized C57Bl/6 mice are shown in **Table 2**. Splenocytes from naïve animals did not react to any of the peptides tested.

**Table 1:** Position and restriction of the YF envelope epitopes in YF-17DD-immunized C57Bl/6 (H2^b^) mice.

YF envelope peptide position	Peptide sequence	Restriction
E1-15	AHCIGITDRDFIEGV	CD8
E201-215	ESWIVDRQWAQDLTL	CD4
E229-243	HHLVEFEPPHAATIR	CD4
E233-247	EFEPPHAATIRVLAL	CD4
E345-359	NKGILVTVNPIASTN	CD4/CD8
E349-363	LVTVNPIASTNDDEV	CD4
E353-367	NPIASTNDDEVLIEV	CD8
E465-479	GINTRNMTMSMSMIL	CD4
E473-487	MSMSMILVGVIMMFL	CD4
E477-491	MILVGVIMMFLSLGV	CD8
E481-493	GVIMMFLSLGVGA	CD8

**Table 2:** Characterization of the cellular responses produced by 17DD vaccination in C57Bl/6 mice.

T-cell responses of C57Bl/6 (H2^b^) mice			
# of IFN-γ SFCs/10^6^ splenocytes	YF envelope 15-mer peptides		
	Total splenocytes	CD8-depleted cells	CD4-depleted cells
**<100**	1–15	201–215	353–367
	201–215	345–359	477–491
	335–367	349–363	481–493
	345–359	473–487	
	349–363		
	473–487		
	477–491		
	481–493		
**100–200**	229–243	229–243	1–15
	465–479	465–479	
**>200**	233–247	233–247	345–359

C57Bl/6 mice were immunized on day 0 and boosted on day 21 with 10^4^ PFUs of the human YF 17DD vaccine, and the splenocytes were tested in IFN-γ ELISPOT assays 7–10 days after the boost. The peptides used for the *in vitro* stimulation were 15-mers overlapping by 11 amino acids and comprising the entire length of the YF envelope protein. The total splenocyte, CD8-depleted splenocyte and CD4-depleted splenocyte populations were analyzed. The SFC values represents the average of two to four experiments performed with pools of three to five mice each.

### p/YFE and pL/YFE DNA immunizations in C57Bl/6 (H2^b^) and BALB/c (H2^d^) mice elicit T-cell responses of similar magnitude and against the same repertoire of epitopes induced by the YF 17DD vaccine

The T-cell responses of H2^b^ and H2^d^ mouse strains induced by immunization with the p/YFE and pL/YFE plasmids were evaluated, and the results were compared with the responses observed for the YF 17DD vaccine immunization. Immunization with the p/YFE or pL/YFE plasmids generated a vigorous T-cell response in C57Bl/6 mice. Both plasmids, in addition to bringing about a T-cell response pattern similar to that produced by the YF 17DD vaccine, were able to elicit a significantly higher number of IFN-gamma SFCs (>200 IFN-gamma SFCs/10^6^ splenocytes) for many immunogenic peptides of the YF envelope protein (**Table 3**; p<0.05) than was immunization with the attenuated virus vaccine.

**Table 3:** Comparison of the C57Bl/6 and BALB/c T-cell responses produced by immunization with p/YFE, pL/YFE and the YF 17DD vaccine.

C57Bl/6 (H2^b^)				BALB/c (H2^d^)			
# of IFN-γ SFCs/10^6^ splenocytes	YF envelope 15-mer peptides (10 μg/mL)			# of IFN-γ SFCs/10^6^ splenocytes	YF envelope 15-mer peptides (10 μg/mL)		
	YF 17DD vaccine	p/YFE	pL/YFE		YF 17DD vaccine	p/YFE	pL/YFE
**<200**	1–15	5–19	169–183	**<200**	25–39	65–79	21–35
	201–215	169–183	201–215		129–143	129–143	25–39
	229–243	201–215	349–363		133–147	137–151	129–143
	233–247	225–239	385–389		157–171	201–215	201–215
	345–359	317–331	413–427		201–215	213–227	213–227
	349–363	345–359	417–431		213–227	233–247	233–247
	465–479	349–363	473–487		221–235	237–251	237–251
	473–487	353–367			233–247	425–439	437–451
	477–491	385–389			237–251	437–451	465–479
					329–343	461–475	477–491
					425–439	461–475	
					437–451	465–479	
					465–479	473–487	
					473–487	477–491	
					477–491		
**200–400**		229–243	1–15	**200–400**	61–75	133–147	61–75
		417–431	229–243				133–147
		473–487	233–247				
		465–479	345–359				
			465–479				
**401–600**		233–247		**401–600**	57–71	57–71	
		413–427				61–75	
**>600**		1–15		**>600**		57–71	

C57Bl/6 and BALB/c mice were immunized on day 0 and boosted on day 21 with 10^4^ PFUs of the human YF 17DD vaccine or with 50 μg of either p/YFE or pL/YFE. Total splenocytes were harvested 7–10 days after the last immunization and assayed *in vitro* using an IFN-γ ELISPOT assay with 15-mers overlapping by 11 amino acids and comprising the entire length of the YF envelope protein. The SFC values represent the average of two to four experiments performed with pools of three to five mice each (p<0.05).

Interestingly, p/YFE was able to generate a considerable response to the E_413–427_ and E_417–431_ peptides, which were not present after YF 17DD immunization and were very scarce after pL/YFE immunization (**Table 3**). Remarkably. p/YFE also brought about a stronger response to peptide E_1–15_ than both the YF 17DD and pL/YFE vaccines.

In BALB/c immunized mice, both the p/YFE and pL/YFE DNA constructs generated an immune response very similar to that obtained with the YF 17DD vaccine, eliciting almost the same immunogenic determinants (**Table 3**). The only considerable exception was the lack of response in YF-17DD-immunized mice to peptide E_329–343_, which contains a previously characterized MHC class I epitope (CD8^+^ response) (**Table 4**). Both the p/YFE and pL/YFE plasmids also produced a significantly higher number of T cells specific to the immunodominant E_57–71_ and E_61–75_ peptides (p<0.05), which contain MHC class I and class II epitopes for the H2^d^ mouse strain. The number of IFN-gamma SFCs was similar for all remaining positive peptides. Groups of mice from both strains immunized with either the empty plasmid or a plasmid expressing only the LAMP protein did not react to any of the peptides tested.

**Table 4:** Position and restriction of the YF envelope epitopes in YF-17DD-immunized BALB/c (H2^d^) mice.

YF envelope peptide position	Peptide sequence	Restriction
E25-39	LEQDKCVTVMAPDKP	CD4
E57-71	RKVCYNAVLTHVKIN	CD8^1^/CD4
E61-75	YNAVLTHVKINDKCP	CD8^1^/CD4
E129-143	EVDQTKIQYVIRAQL	CD4^2^
E133-147	TKIQYVIRAQLHVGA	CD4^2^
E157-171	KTLKFDALSGSQEVE	CD4
E201-215	ESWIVDRQWAQDLTL	CD4
E213-227	LTLPWQSGSGGVWRE	CD4
E221-235	SGGVWREMHHLVEFE	CD4
E233-247	EFEPPHAATIRVLAL	CD4
E237-251	PHAATIRVLALGNQE	CD4
E329-343	PCRIPVIVADDLTAA	CD8
E425-439	GFFTSVGKGIHTVFG	CD4
E437-451	VFGSAFQGLFGGLNW	CD4
E461-475	LIWVGINTRNMTMSM	CD4
E465-479	GINTRNMTMSMSMIL	CD4
E473-487	MSMSMILVGVIMMFL	CD4
E477-491	MILVGVIMMFLSLGV	CD4

^1^ These peptides contained class I immunodominant epitopes and secondary class II epitopes.

^2^ These peptides contained class II immunodominant epitopes (Maciel et al., 2008).

### Evaluation of the humoral responses generated by the YF 17DD and the DNA construct immunizations

The protection provided by the YF vaccine is mainly attributed to the neutralizing antibody (nAb) response generated after vaccination. Because the presence of nAb is a hallmark of protection, we evaluated the humoral response of C57Bl/6 and BALB/c mice after immunization with the DNA constructs and compared them with the levels of nAb obtained after immunization with the YF 17DD vaccine. To investigate the kinetics of nAb responses, the animals were immunized at days zero, 30 and 45 and bled 15 days after each immunization.

**Fig 4:**
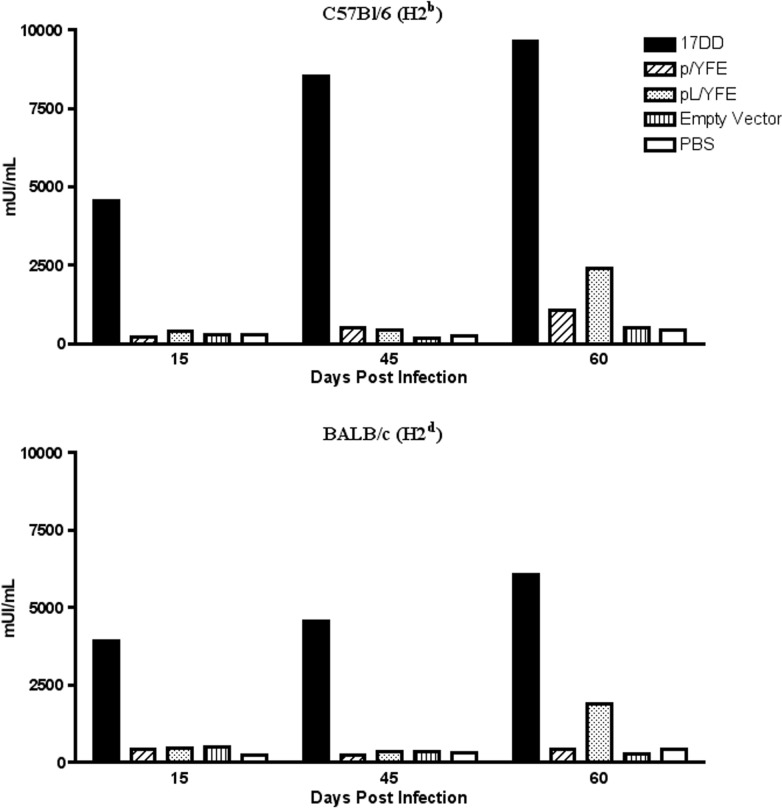
Kinetics of the neutralizing antibody levels in C57Bl/6 and BALB/c mice. C57Bl/6 and BALB/c mice were immunized with the YF 17DD vaccine, the DNA constructs or PBS on days 0, 30 and 45, and sera were obtained on days 15, 45 and 60. Sera were tested individually in neutralization assays and compared with a standard monkey serum having a known concentration of anti-YF neutralizing antibodies. The figures represent 1 of 3 experiments, which each had similar results. Bars represent the mean ± SE of 3–14 mice/group.

The YF 17DD vaccine was able to produce very high levels of nAb in C57Bl/6 and BALB/c mice after the first immunization (day 15). nAb levels in C57Bl/6 mice seemed to reached a plateau after the second immunization (day 45) and increased slightly after the third immunization (day 60), whereas BALB/c mice showed increasing levels of nAb after the second (day 45) and third (day 60) immunization with the YF 17DD vaccine. The levels of nAb observed in C57Bl/6 mice (≥9,664.0 mIU/mL; obtained at the highest dilution tested) were approximately 20% higher compared with the levels observed in BALB/c mice (7,500±780.1 mIU/mL) (**Fig 4**). In C57Bl/6 mice, both plasmids expressing the whole envelope protein (p/YFE and pL/YFE) were able to produce significant levels of nAb (p<0.0039 and p<0.002, respectively) after three immunizations compared with the empty vector control. The DNA plasmid pL/YFE, expressing the chimeric E-LAMP protein, led the BALB/c mice to produce higher titers of nAb after the second immunization compared to the p/YFE plasmid. The levels of nAb titers increased after the third immunization and were significantly higher (p<0.045) than those of the control groups immunized with empty vector or PBS (**Fig 4**).

On average, the pL/YFE DNA immunization elicited nAb titers 7-fold greater than the p/YFE DNA immunization. Compared with the 17DD attenuated virus vaccine, the nAb titers produced by the pL/YFE DNA vaccine were approximately 3.5-fold lower. The fact that these DNA vaccines produced these levels of nAb may still be considered significant.

### Protection evaluation via challenge

**Fig 5:**
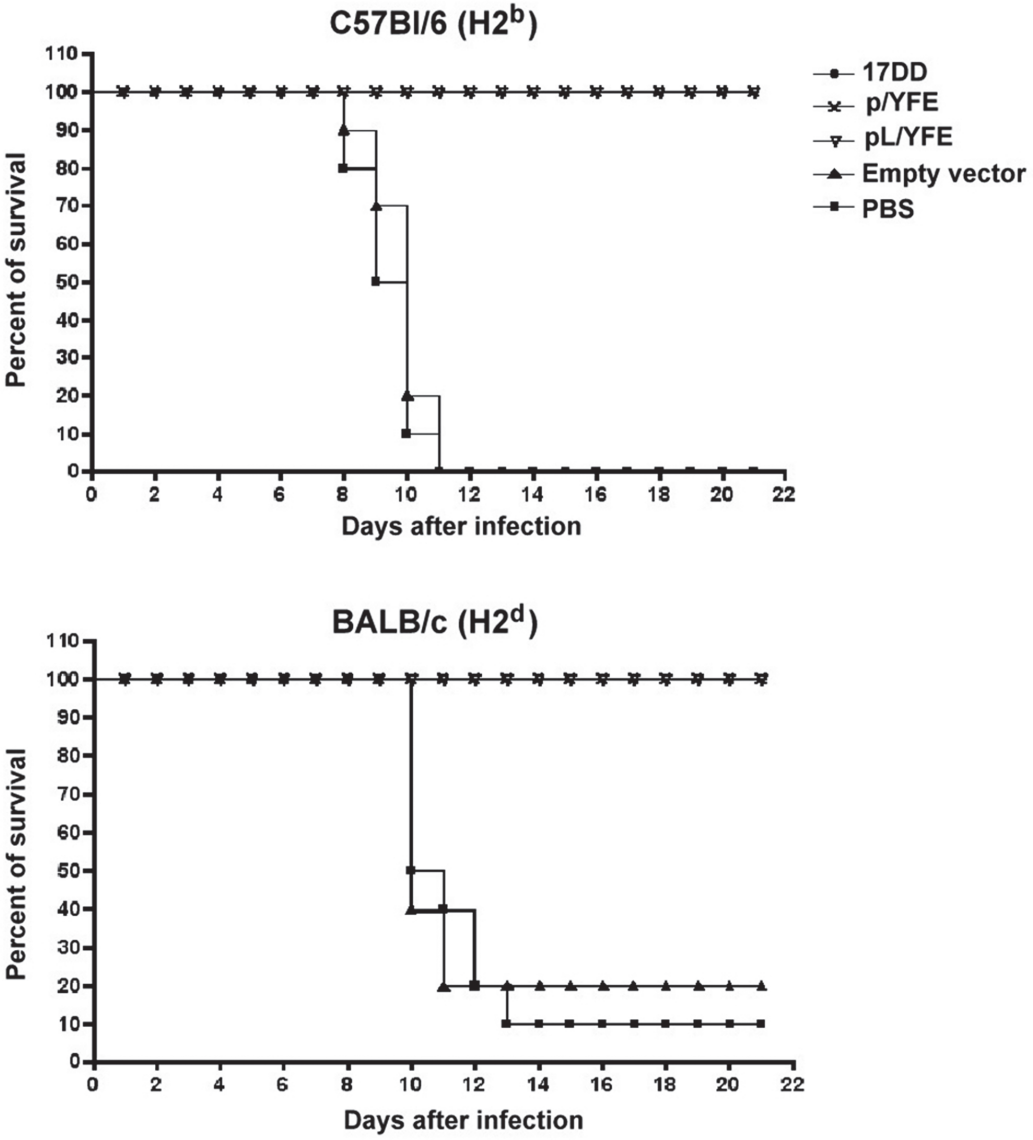
Survival curves for C57Bl/6 and BALB/c mice after challenge experiments. C57Bl/6 and BALB/c immunized mice were challenged with 10^5^ PFUs of the YF 17DD virus, administered via intra-cerebral injection. Both mouse strains were completely protected by the 17DD and both DNA-based (p/YFE and pL/YFE) vaccines. Overall, C57Bl/6 mice were considered more susceptible than BALB/c mice because C57Bl/6 mice in the negative control groups (empty vector and PBS) began to die 8 days after the challenge and all animals were dead by day 12, whereas the BALB/c mice in the negative control groups began to die 10 days after the challenge and very few animals were still alive after the challenge experiment.

Intra-cerebral challenge with the YF 17DD virus in mice is a useful model for evaluating the protection provided by vaccine candidates [34]. We evaluated our DNA constructs using the immunization/challenge model by injecting 10^5^ PFUs of the YF 17DD virus intra-cerebrally into DNA-immunized C57Bl/6 and BALB/c mice. The animals were immunized three times at days 0, 30 and 45 and were challenged 15 days after the last immunization. As previously reported [35], immunization with YF 17DD vaccine was able to protect both C57Bl/6 and BALB/c mouse strains against the intra-cerebral challenge. In a similar fashion, immunization with both DNA constructs expressing the full-length YF envelope protein was able to fully protect both mouse strains from the lethal challenge. The majority of mice immunized with PBS or empty vector died 10 to 14 days after the challenge assay (**Table 5** and **Fig 5**).

**Table 5:** C57Bl/6 and BALB/c mouse challenge experiments after immunization with the YF 17DD vaccine or the DNA constructs.

Immunization	Challenge (10^5^ PFUs)	C57Bl/6		BALB/c	
		Mortality (deaths/ total number inoculated)	Average survival time(days ± SD)	Mortality (deaths/ total number inoculated)	Average survival time(days ± SD)
YF 17DD vaccine	YF 17DD virus	0/10	-	0/10	-
p/YFE	YF 17DD virus	0/10	-	0/10	-
pL/YFE	YF 17DD virus	0/10	-	0/10	-
Empty vector	YF 17DD virus	10/10	12.8±0.92	8/10	10.3±0.46
PBS	YF 17DD virus	10/10	12.4±0.97	9/10	10.9±1.17
PBS	PBS	0/5	-	0/5	-

## Discussion

YF infection continues to be a worldwide problem, especially in tropical areas [11], but this may change as the world continues to be affected by climate change. Despite the high efficiency of commercially available YF vaccines, 17D and 17DD, there are a few reports of rare but fatal side-effects after vaccination [9, 11, 12]. Furthermore, these vaccines are not recommended for infants, pregnant women, immunodeficient subjects, or those allergic to the egg components present in the vaccine formulations [36]. In light of these factors, there is reason to pursue complementary or alternative YF vaccine strategies that could replace the use of the virus-attenuated vaccine version.

Although no DNA vaccines have yet been approved for human use, they represent potential candidates to replace live/attenuated vaccine formulations because they are considered safer. DNA formulations can be easily manipulated, do not require a cold-chain for distribution and eliminate the infectious nature of live/attenuated agents. They also allow the manipulation of immunogens to provide the immune system with the desired epitopes and signals while avoiding the use of unnecessary or potentially harmful antigens or epitopes [37, 38].

Previous studies have described the development of DNA vaccines against flaviviruses based on the expression of the pM/M-E virus sequence cloned in-frame with the LAMP sequence [17, 22, 24]. This approach showed that the chimeric protein, driven by the cytoplasmic sequence of LAMP, was targeted to LAMP-containing organelles, which also co-localized with MHC-class-II-rich intracellular compartments [25]. The immunofluorescence microscopy study of our plasmid expressing the YF pM/M-E in-frame with LAMP (pL/YFE) produced findings similar to those of previous studies and suggests that the presence of LAMP was indeed able to lead the chimeric protein to lysosomes; in contrast, the expression of pM/M-E without LAMP (p/YFE) resulted in a reticular membrane distribution. We also investigated the immunogenicity of these two plasmids as DNA vaccines against YF virus infection. The performance of our DNA constructs was compared with the successful human YF 17DD vaccine, which is a better positive control than the inactivated virus emulsified in CFA, for example [17, 22], which is used when an approved vaccine is not available.

We first carried out epitope mapping of the E protein, comparing the 17DD vaccine with the p/YFE and pL/YFE DNA constructs. We observed that the epitope profile repertoire recognized by the T cells of mice immunized with the DNA constructs was very similar to that of mice immunized with the standard YF vaccine. Moreover, the majority of the T-cell responses in the DNA-immunized mice showed higher numbers of IFN-gamma SFCs compared with the numbers observed for the 17DD vaccine. The immunization of C57Bl/6 mice with the DNA constructs expressing the whole envelope protein resulted in recognition of three extra peptides that were not produced by the YF 17DD vaccine. The T-cell response against the peptide E_169–183_ generated by p/YFE and pL/YFE was significant, and the responses to the peptides E_413–427_ and E_417–431_ were even higher in mice immunized with p/YFE compared with mice immunized with pL/YFE.

It seems that DNA immunization was able to lead to the presentation of some additional epitopes that are not normally induced by the YF 17DD vaccine. It is possible that different antigen-presenting cells (APCs) processed different epitopes, according to the source of the antigen, i.e., attenuated virus or DNA plasmids. However, the lack of response to the E_329–343_ 15-mer observed in BALB/c mice immunized with DNA seemed to be partially because the same cells were able to respond to the minimum epitope within that sequence, as seen against the 9-mer E_330–338_. Our results also expanded the C57Bl/6 (H2^b^) epitope mapping for the envelope protein of the YF virus. Sequences E_1–15_ and E_233–247_ were previously described as containing CD8 and CD4 epitopes, respectively [39]; however, we were able to identify several new epitopes, six for CD4 and four for CD8 (**Table 2**).

The presence of anti-YF nAb is a recognized hallmark of protection against YF infection. A dose of 10^4^ PFUs of YF 17DD virus was potent enough to produce a high concentration of nAb after a single immunization in both mouse strains. The plasmid expressing the chimeric E-LAMP protein (pL/YFE) was able to produce significantly higher concentrations of anti-YF nAb after three immunizations in both mouse strains compared with the controls or p/YFE; however, the levels of nAb were considerably lower compared with the YF 17DD immunization. The p/YFE plasmid, expressing only the YF E protein, failed to generate high levels of anti-YF nAb in BALB/c mice and produced only a modest increase of anti-YF nAb in C57Bl/6 mice. These data are in accordance with previous reports demonstrating that the expression of chimeric proteins in-frame with LAMP lead to an improvement in B-cell responses [17, 22, 30]. Others have used extended immunization protocols to produce higher levels of antibody [17]. Although we have not tested this hypothesis here, it is interesting to speculate that extra DNA immunizations could further increase the levels of anti-YF nAb observed.

In addition to nAb, complement-fixing antibodies have also been described as a protective mechanism against YF [40, 41]. In fact, it has been shown in an animal model that expression of the YF NS1 protein in the vaccinia virus could partially protect mice from an intracranial challenge [42], most likely through a mechanism involving complement-fixing antibodies. We cannot rule out the hypothesis that immunization with the YF envelope protein, as a DNA plasmid, could lead to the presentation of B-cell epitopes different from those found after immunization with the attenuated YF vaccine. Moreover, these epitopes could be sites for neutralizing and complement-fixing antibodies. Thus, the characterization of the B-cell epitopes in the context of DNA vaccines could potentially become a relevant parameter for comparing DNA vaccines to their virus-attenuated counterparts.

To further explore the protection provided by DNA immunization, we challenged immunized mice with an intracranial injection of the 17DD virus. This *in vivo* protection assay enables the evaluation of how effectively a vaccine candidate can prevent the encephalitis caused by infection with the YF virus, and it has been extensively used [35, 41–44]. Both DNA constructs, p/YFE and pL/YFE, were able to protect both mouse strains from an intracranial challenge. These two plasmids were able to promote a very similar profile of T-cell epitope recognition compared to the YF 17DD vaccine. However, only the pL/YFE was able also to produce significant levels of anti-YF nAb. It is possible that the p/YFE plasmid, which did not raise appreciable levels of anti-YF nAb, led to the protection of the challenged mice through complementary mechanisms in the presence of low levels of nAb. It is also possible that strong T-cell responses were able to mediate protection in this system. T cells may play a role in protection from encephalitis caused by flaviviruses; it has been demonstrated that the depletion of CD4^+^ and/or CD8^+^ lymphocytes leads to a decrease in the protection offered by an experimental vaccine expressing the dengue envelope protein in the context of the YF virus [39]. The possibility that these DNA vaccines provide T-cell mediated protection is very interesting and we are planning to investigate this in more detail.

The results reported here are very encouraging, and we are confident that this vaccine candidate is worth further investigation in more relevant animal models, specifically in a non-human primate challenge model. It is interesting that even with lower neutralizing antibody titers the DNA vaccine was still capable of protection, suggesting an important role for T-cell mediated protection. In further studies, we plan to dissect in more details the mechanisms of protection provided by these DNA vaccines. Another critical point is the duration of the protection, and this also needs to be addressed in more relevant animal models. In summary, this research shows that expression of the envelope protein in-frame with the cytoplasmic targeting sequence of LAMP led to high levels of anti-YF nAb and produced a strong T-cell response. The possibility of generating a protective anti-YF response through a DNA vaccine may provide a safer alternative to the attenuated YF virus vaccine and should be further investigated.
